# Saudi views on consenting for research on medical records and leftover tissue samples

**DOI:** 10.1186/1472-6939-11-18

**Published:** 2010-10-18

**Authors:** Mohammad M Al-Qadire, Muhammad M Hammami, Hunida M Abdulhameed, Eman A Al Gaai

**Affiliations:** 1 Center for Clinical studies and Empirical Ethics, King Faisal Specialist Hospital and Research Centre, P O Box # 3354 (MBC 03), Riyadh 11211, Saudi Arabia

## Abstract

**Background:**

Consenting for retrospective medical records-based research (MR) and leftover tissue-based research (TR) continues to be controversial. Our objective was to survey Saudis attending outpatient clinics at a tertiary care hospital on their personal preference and perceptions of norm and current practice in relation to consenting for MR and TR.

**Methods:**

We surveyed 528 Saudis attending clinics at a tertiary care hospital in Saudi Arabia to explore their preferences and perceptions of norm and current practice. The respondents selected one of 7 options from each of 6 questionnaires.

**Results:**

Respondents' mean (SD) age was 33 (11) years, 42% were males, 56% were patients, 84% had ≥ secondary school education, and 10% had previously volunteered for research. Respectively, 40% and 49% perceived that the norm is to conduct MR and TR without consent and 38% and 37% with general or proposal-specific consent; the rest objected to such research. There was significant difference in the distribution of choices according to health status (patients vs. companions) for MR (adjusted Kruskal-Wallis test P = 0.03) but not to age group, gender, education level, or previous participation in research (unadjusted P = 0.02 - 0.59). The distributions of perceptions of current practice and norm were similar (unadjusted Marginal Homogeneity test P = 0.44 for MR and P = 0.89 for TR), whereas the distributions of preferences and perceptions of norm were different (adjusted P = 0.09 for MR and P = 0.02 for TR). The distributions of perceptions of norm, preferences, and perceptions of current practice for MR were significantly different from those of TR (adjusted P < 0.009 for all).

**Conclusions:**

We conclude that: 1) there is a considerable diversity among Saudi views regarding consenting for retrospective research which may be related to health status, 2) the distribution of perceptions of norm was similar to the distribution of perceptions of current practice but different from that of preferences, and 3) MR and TR are perceived differently in regard to consenting.

## Background

Obtaining individual consent is the default for human subject research involving individually identifying data [1]. However, retrospective research on medical records (MR) or leftover tissues (TR) may be ethically conducted without the explicit consent of individual subjects under certain conditions; where individual interest of confidentiality is outweighed by the expected utility of the research [2,3]. Obtaining the consent of individuals before the use of their medical records can be an obstacle to research [4,5] and a wider debate on the utilization of medical records in medical research has been advocated [6].

Several empirical ethics studies on consenting for retrospective research have been published [7-14]. However, none has been conducted in Arabic/Islamic countries, directly compared attitudes toward MR and TR, or differentiated between preference (a statement about the person who has the preference) and perception of norm (a statement about the thing which is being judged).

The aim of the current study was to obtain empirical evidence from Saudis attending the outpatient clinics at a tertiary care hospital on personal preferences and perceptions of norm and current practice in relation to consenting for MR and TR.

## Methods

Five hundred sixty seven adult individuals who were present in the outpatients' clinics of the King Faisal Specialist Hospital and Research Centre (KFSH&RC), a tertiary care hospital and research center in Riyadh, Kingdom of Saudi Arabia, between May 2007 and January 2008 were invited to participate in the study. The number of individuals invited from each clinic area was prorated based on average clinic load. The sample size and sampling method were convenience-based. We aimed to have 500 evaluable respondents, expecting a refusal/ineligibility rate of about 20%. Potential participants were individually and consequently approached; and were enrolled if they were able to understand the study and agreed to participate.

Six questionnaires were developed by the authors based on literature and clinical research regulations review. After initial development, they were presented for comments to 3 KFSH&RC Research Ethics Committee members and revised accordingly. Face validity was confirmed by interviewing 5 participants after completing the questionnaires. The final version was pilot-tested on 10 patients and 10 patient's companions, and found clear and stable (over 2-4 days). Questionnaires one and two address personal preference, questionnaires three and four address perception of norm (the rules that should be followed, regardless of what is personally preferred), and questionnaires five and six address perception of the current practice at KFSH&RC. Questionnaires one, three, and five are on MR and questionnaires two, four, and six are on TR. The questionnaire on personal preference in relation to consenting for MR is shown in Table 1. Equivalent statements were used in the other questionnaires with appropriate modifications. For example, for the first three statements, we used the phrase "I prefer that" combined with "is not" and "my/I" to indicate personal preference and "I think that" combined with "should not be" and "patient" to indicate perception of norm. For perception of current practice questionnaires, "I prefer that" was omitted and "is not" was combined with "patient". For TR questionnaires, "the information in my medical records" was replaced by "my leftover tissues". The statements in each questionnaire were arranged from most to least restrictive (statement # 1 to 7). Before completing the questionnaires, participants were given introductory information on the use of identifiable clinical data/tissue in research. The questionnaires were administered by the investigators in a structured interview in Arabic language. For each questionnaire, the participants were asked to choose the most representative statement. The six questionnaires together with the introductory information given to participants (English version, confirmed by back translation) are available in the Additional file 1.

**Table 1:** Questionnaire on the preferred consenting for medical records research

I - What I personally prefer: Medical Records: (choose one answer)
1) I prefer that the information in my medical records is not used except for my medical care.
2) I prefer that the information in my medical records is not used in medical research unless I gave my prior consent for each type of research.
3) I prefer that the information in my medical records is not used in medical research unless I gave a general consent for research.
4) It is ok to use the information in my medical records in medical research if approval of the research ethics committee was obtained; there is no need for my consent.
5) It is ok to use the information in my medical records in medical research without my consent or the approval of the research ethics committee as long as the researcher is affiliated with KFSH&RC.
6) It is ok to use the information in my medical records in medical research without my consent or the approval of the research ethics committee as long as the researcher is Saudi.
7) It is ok to use the information in my medical records in medical research without my consent or the approval of the research ethics committee as long as the researcher is scientifically competent and regardless to his/her nationality.

Data on the following were collected: age, gender, patient (individuals who came for a clinic appointment) or patient's companion (individuals who are accompanying a relative or friend who came for a clinic appointment), previous participation in research, and educational level (illiterate -did not have formal education, primary, secondary, Bachelors, Master or PhD). For analysis purposes, respondents were divided into 5 age groups (18-29, 30-39, 40-49, 50-59, and ≥ 60 years).

The number (%) of respondents who chose one of the 7 statements (for each of the 6 questionnaires) was determined. We used Chi^2 ^test to examine the null hypothesis of random distribution of choices of statements for each questionnaire. We conducted 30 Kruskal-Wallis tests (null hypothesis of no association) for subgroups analysis, a P value of ≤ 0.0017 (0.05/30) was considered significant at the 0.05 level [15]. We also conducted 9 Marginal Homogeneity tests comparing the 6 questionnaires, a P value < 0.006 (0.05/9) was considered significant at the 0.05 level [15]. In addition, we examined the correlation between individual scores for each questionnaire using Spearman's test. SPSS Statistics 17.0 (2008) for Windows was used for data analysis.

The study was conducted in accordance with the ethical principles contained in the Declaration of Helsinki (2008) after approval of the Research Ethics Committee of KFSH&RC. All respondents gave verbal consent.

## Results

Out of the 567 subjects invited to participate in the study, 36 refused and 3 were excluded by the investigators because they could not demonstrate adequate understanding of the study. Table 2 shows the demographics of the 528 participants.

**Table 2:** Characteristics of the 528 Participants

	M (SD)	N (%)
Age, year	33 (11)	
Age group, year:		
18-29		220 (42%)
30-39		170 (33%)
40-49		81 (16%)
50-59		33 (6%)
≥60		14 (3%)
Gender:		
Male		223 (42%)
Female		305 (58%)
		
Education Level:		
Illiterate		23 (4%)
Primary		63 (12%)
Secondary		189 (36%)
Bachelors		237 (45%)
Master or PhD		16 (3%)
		
Previous Participation in Research:		
Yes		53 (10%)
No		475 (90%)
		
Health Status:		
Patient		293 (56%)
Companion		235 (44%)

### Consenting choices for the use of medical records and leftover tissue in research

Table 3 shows the number (%) of participants who chose one of the seven statements regarding consenting for MR and TR from one of three perspectives: perception of norm, personal preference, and perception of current practice at KFSH&RC. Each questionnaire was completed by at least 95% of the participants. Respectively, 40% and 49% of participants perceived that the norm is to conduct MR and TR without consent (statements 4 to 7), 38% and 37% with general or proposal-specific consent (statement 2 and 3), and 24% and 30% without ethics committee approval (statements 5 to 7). In contrast, 23% and 14% thought that medical records and leftover tissues, respectively, should be used only for medical care (statement 1). The least chosen (by 3.3% and 2.0%, respectively) was statement # 6 which states that MR and TR can be conducted without consent or research ethics committee approval as long as the researcher is Saudi.

**Table 3:** Choices of consenting for medical records research (MR) and leftover tissue research (TR) from the perspective of perception of norm (norm), personal preference (preference), and perception of current practice (practice)

Statement*								
	1 = No research use	2 = Proposal specific consent	3 = General research consent	4 = No consent, only REC	5 = No consent or REC, if institution's researcher	6 = No consent or REC, if Saudi researcher	7 = No consent or REC, if competent researcher	Total
**MR**								
Norm ^a,1^	116 (22.8)	104 (20.5)	87 (17.1)	81 (15.9)	30 (5.9)	17 (3.3)	73 (14.4)	508
Preference ^b,1^	88 (17.0)	98 (18.9)	94 (18.2)	109 (21.0)	41 (8.0)	18 (3.5)	70 (13.5)	518
Practice ^c^	115 (23.0)	77 (15.4)	81 (16.2)	105 (21.0)	50 (10.0)	13 (2.6)	60 (12.0)	501
**TR**								
Norm ^a,2^	72 (14.2)	97 (19.2)	90 (17.8)	97 (19.2)	49 (9.7)	10 (2.0)	91 (18.0)	506
Preference ^b,2,3^	55 (10.7)	89 (17.3)	81 (15.7)	109 (21.2)	64 (12.4)	14 (2.7)	103 (20.0)	515
Practice ^c,3^	86 (17.2)	75 (15.0)	69 (13.8)	113 (22.6)	80 (16.0)	15 (3.0)	63 (12.6)	501

*See Table 1 for full description of each statement. REC, Research Ethics Committee. Data indicate the number (%) of participants who chose the corresponding statement. Chi square test for the null hypothesis of random distribution was significant (P < 0.001) for each of the six questionnaires. Using Marginal Homogeneity test, perception of norm, preference, and perception of current practice were compared for MR and TR; and MR and TR were compared in relation to perception of norm, preference, and perception of current practice.

^a, b, c ^Adjusted P < 0.009.

^1^Adjusted P = 0.09.

^2^Adjusted P = 0.02.

^3^Adjusted P = 0.05. For the rest of comparisons, unadujsted P = 0.44-0.89

### Is there a difference between personal preference and perception of norm?

We compared the distribution of participants' preferences to the distribution of what they perceived as the norm (Table 3). We found significant difference for TR but not for MR (adjusted P = 0.02 and P = 0.09, respectively). For both TR and MR, the distribution was relatively shifted to the left, indicating stricter consenting requirements for preferences. On the other hand, significant correlations of individual scores were found between personal preference and perception of norm (r = 0.46, P < 0.001 for MR and r = 0.49, P < 0.001 for TR), indicating a high degree of intra individual consistency.

### Is there a difference between perception of norm and perception of current practice?

We did not find a significant difference (Table 3) between the distribution of participants' perceptions of norm and current practice for MR (unadjusted P = 0.44) or TR (P = 0.89). We found significant correlations in individual scores between perceptions of norm and current practice (r = 0.28, P < 0.001 for MR and r = 0.31, P < 0.001 for TR).

### Is there a difference between personal preference and perception of current practice?

We did not find a significant difference (Table 3) between the distribution of participants' preferences and perceptions of current practice for MR (unadjusted P = 0.16) or TR (adjusted P = 0.05). We found significant correlations in individual scores between personal preference and perception of current practice (r = 0.27, P < 0.001 for MR and r = 0.25, P < 0.001 for TR).

### Is there a difference in consenting choices for MR and TR?

We compared the distributions of the consenting choice for MR and TR, from the three perspectives (Table 3). The differences in distribution of choices for the two types of retrospective research were statistically significant (adjusted P < 0.009) for personal preference, perception of norm, and perception of current practice. Overall, stricter requirements were chosen for MR than for TR (relative left shift in MR distribution). On the other hand, we found strong correlations of individual scores between the two types of retrospective research (r = 0.67-0.75, P < 0.001 for each of the three perspectives), indicating a high degree of intra-individual consistency.

### Association between consenting choice and participants' characteristics

We investigated whether there are differences in the distribution of consenting choice among subgroups defined by age, gender, educational level, previous research experience, or health status (patient or companion). There were no significant differences among the age groups (unadjusted P = 0.20 to 0.99), genders (unadjusted P = 0.009 for TR preference to 0.49), previous research experience (unadjusted P = 0.14 to 0.83), educational level (unadjusted P = 0.02 for MR perception of norm to 0.97), or health status (unadjusted P = 0.10 to 0.88), except for perception of norm for MR (adjusted P = 0.03). Table 4 shows the distribution of perceptions of norm for MR and TR according to health status.

**Table 4:** Choices of consenting for medical records research (MR) and leftover tissue research (TR) from the perspective of perception of norm according to health status

Statement*								
	1 = No research use	2 = Proposal specific consent	3 = General research consent	4 = No consent, only REC	5 = No consent or REC, if institution's researcher	6 = No consent or REC, if Saudi researcher	7 = No consent or REC, if competent researcher	Total
**MR**								
Patients ^a,1^	71 (26.1)	64 (23.5)	47 (17.3)	40 (14.7)	11 (4.0)	12 (4.4)	27 (9.9)	272
Companions ^b,1^	45 (19.1)	40 (16.9)	40 (16.9)	41 (17.4)	19 (8.1)	5 (2.1)	46 (19.5)	236
**TR**								
Patients ^a, 2^	41 (15.3)	55 (20.5)	50 (18.7)	50 (18.7)	25 (9.3)	6 (2.2)	41 (15.3)	268
Companions ^b, 2^	31 (13.0)	42 (17.6)	40 (16.8)	47 (19.7)	24 (10.1)	4 (1.7)	50 (21.0)	238

*See Table 1 for full description of each statement. REC, Research Ethics Committee. Data indicate the number (%) of participants who chose the corresponding statement. MR was compared to TR using Marginal Homogeneity test, and patients were compared to companions using Kruskal-Wallis test.

^a ^Unadjusted P < 0.001.

^b ^Unadjusted P = 0.004.

^1 ^Unadjusted P = 0.001.

^2^Unadjusted P = 0.10.

The significant association between perception of norm and health status was examined further. The patients subgroup had a mean (SD) age of 35 (11) years, 32% males, 81% with ≥ secondary school education, and 8% with previous research experience. The companions subgroup had a mean (SD) age of 31 (9) years, 54% males, 89% with ≥ secondary school education, and 11% with previous research experience. The difference in the distribution of choices between the two types of retrospective research continued to be present in both subgroups (unadjusted P < 0.001 for MR and TR preference, MR perception of norm, and TR perception of current practice, P = 0.004 for TR perception of norm, and P = 0.01 for MR perception of current practice). The differences in distributions of preferences and perceptions of norm also continued to be present (unadjusted P = 0.001 for MR and P = 0.003 for TR) in the patients subgroup but not in the companions subgroup (unadjusted P = 0.92 for MR and 0.16 for TR), suggesting that most of the difference in the entire group was contributed by the patient subgroup. In contrast, the difference in the distributions of preferences and perceptions of current practice for TR continued to be present in each subgroup (unadjusted P = 0.05 for both groups), suggesting that both subgroups contributed to the difference in the entire group.

## Discussion

Consenting for retrospective medical records-based research (MR) and leftover tissue-based research (TR) continues to be controversial. The Utilitarian approach, aiming to safeguard scientific integrity of data, maintains that an implicit consent should be adequate (opt-out approach). The Rights approach that emphasizes autonomy and confidentiality demands an explicit consent (opt-in approach) [7]. In prospective studies, investigators can readily seek verbal or written consent [16]. In retrospective studies, however, participants might not be practically contactable and contacting them might cause distress [1]. Research Ethics Committees review may help maintain confidentially of data by permitting only certain research without patients' consent [17].

Clinical research centers in Saudi Arabia have, to a large extent, individually adopted Western and international regulations of clinical research such as the US Title 45 Code of Federal Regulation part 46 [3], the Declaration of Helsinki [18], and the guidelines of the Council for International Organizations of Medical Sciences [19]. These regulations permit the Research Ethics Committees to waive the requirement of informed consent for certain types of retrospective research. Although the Research ethics Committees may be guided by theoretical ethical arguments, they remain uninformed about public views on consenting, as empirical ethics studies have not been conducted in this or similar culture.

This survey was conducted to explore the views of Saudis on consenting for MR and TR in the setting of a tertiary care hospital. The views were examined from three perspectives: personal preference, perception of norm, and perception of current practice. The population surveyed was rather balanced (56% patients, 44% companions, from a variety of outpatient clinics). We found that: 1) there is a considerable diversity among Saudi views regarding consenting for retrospective research which may be related to health status, 2) MR and TR are perceived differently in regard to consenting, and 3) the distribution of perceptions of norm was similar to the distribution of perceptions of current practice but different from that of preferences.

We found a large variation in the perceived norm for consenting for medical records and leftover tissue-based research. Respectively, only 38% and 37% of participants required a consent (general or proposal-specific), the rest either did not require a consent (40% and 49%) or believed that medical records and leftover tissue should be used only for personal medical care (23% and 14%). In comparison, 60% of the Canadian public felt that their permission should be obtained before their health information can be used for research [10]. Respectively, 65.8% and 29% of Americans required consenting for research on clinically derived and research derived personally identifying samples [13]. In another American study on a special group with serious medical conditions, the majority of respondents (55.4%) disagreed to the use of their medical records for research purposes without their permission [9]. Finally, a Swedish study showed that 72% of participants thought that a general consent to use stored samples for new studies is enough provided an approval by a research ethics committee [14]. In this study, participants who required consenting were divided equally between a one-time general consent choice and a proposal-specific consenting choice. To our knowledge, this is the first study to directly compare these two choices of consenting.

We found that although only about 37% of participants required consent, most participants (> 77%, statements 2-7) do not, in principle, oppose retrospective research. This is consistent with findings from other studies [11,12,20]. About 60% and 85% of New Zealanders were willing (answered yes or maybe) to share their identifiable and non-identifiable health information, respectively, with health researchers [11]. Similarly, American respondents' assent rate for storage and future use of left over samples for research was 89.4% and 97.3%, using a simple statement or a more detailed one time general consent format, respectively [12]. A review article, examining individual preferences for TR consenting options, also found high willingness (79-95%) to provide one-time general consent [20].

This study was specifically designed to compare perception of norm, personal preference, and perception of current practice; and to compare views toward MR and TR. The distributions of personal preferences and perceptions of norm were significantly different for TR and of borderline significant difference for MR; although a rather strong correlation was found between the distributions of both MR and TR. At least on face value, this speaks against norm-compliant society or a low inclination to express preferences that are different from perceived norm (social desirability bias). There are two types of norm, injunctive norm (people's perception of what is commonly approved or disapproved within a specific culture) and descriptive norm (people's perception of what is commonly done). Although the questionnaires were designed to explore the perception of injunctive norm (the rules that should be followed, regardless of what I personally prefer), it is possible that some respondents confused injunctive norm with descriptive norm. This possibility is supported by the finding that there was no significant difference between perceptions of norm and perceptions of current practice, the fact that "current practice" referred to KFSH&RC, a premier center in the area, and the finding that most of the differences between preferences and perceptions of norm in the entire study population may be contributed by the patients subgroup (who have closer knowledge of "current practice"). The strong correlation between perception of norm and preferences suggests that the disagreement between the two is a matter of degree rather than of direction. It appears that the distribution of choices was "shifted to the left", i.e. stricter, for perceptions of norm compared to preferences, indicating a trusting/satisfied population.

On the other hand, Saudis appeared to be more concerned about MR than TR, with the distribution being relatively shifted to the left for MR. The differences between MR and TR were significant from all three perspectives, despite strong correlations between individual scores. This is rather expected as the information in medical records are more profound, more readily identifying, and more related to patient-comprehended information, than the information that can be obtained from leftover tissues. However, this may indicate less awareness of the potential wealth of information in leftover tissues as a source of DNA, which can identify the tissue source even with fully anonymized samples, is a diary of the future, and relates not only to tissue source but also to his/her family. The importance of privacy and confidentiality are well established in Islamic teachings. For example, Al-Qur'an says, "O ye who believe! enter not houses other than your own, until ye have asked permission and saluted those in them: that is best for you, in order that ye may heed (what is seemly). If ye find none in the house, enter not until permission is given to you: if ye are asked to go back, go back: that makes for greater purity for yourselves: and Allah knows well all that ye do."(Chapter 24, verses 27-28), and prophet Muhammad has said, "if you hear from someone something that he does not wish to be mentioned of him then it is a trust, even if he does not request that it is not mentioned." (Musnad Ahmad, Musnad Al-Kabael 26237)

The distribution of consenting choices was not related to age group, gender, education level, or previous research participation. A British study indicated that the proportion of participants who were not concerned about invasion of privacy varied significantly according to cultural background, ethnicity, and socioeconomic status [7]. However, consistent with our findings, other studies showed that education had no effect on consenting choices [10,13]. It may be expected that sick patients looking for innovative treatment are less strict in consenting requirements. Nevertheless, in one study, the majority of patients with several medical conditions disagreed to use their medical records for research without their permission [9].

We found that the apparent health status was significantly associated with perception of norm for MR. The choices of the patients were shifted to the left compared to the choices of companions for both MR and TR, which indicates that studies that include patients only may overestimate the desired strictness' in consenting of the public. Companionship was defined in our study as not having a clinic appointment at the time of the study. Since some of the companions are likely to be also patients, it would be expected that the observed difference in consenting choices according to health status may be an underestimate. It was observed that studies restricted to population with serious medical or genetic conditions may be biased toward more favorable attitudes towards research [9,12]. Together with our findings, this suggests that although patients may have stricter consenting requirements, the seriously ill who are looking for breakthroughs may be less strict.

The limits of our study include being based on a single hospital and on perception and preferences rather than on actual consenting/participation. Also, the study did not compare identifiable and anonymized data. Current regulations do not require consenting for the use of existing anonymised data in research (it is not considered human subjects research or is exempted from review). Objection to the use of anonymised data in research is expected to be related to objection to research in general or to the purpose of particular research, whereas objection to the use of identifiable data could be related to concerns about privacy and exposure to the risk of harm, in addition. Our study was designed to explore consenting views on identifiable data; these views may be less strict for anonymised data.

## Conclusions

In conclusion, we found that 1) there is a considerable diversity among Saudi views regarding consenting for retrospective research which may be related to health status (overall stricter requirements were expressed by patients), 2) MR and TR are perceived differently in regard to consenting (overall stricter requirements were expressed for MR), and 3) the distribution of perceptions of norm was similar to the distribution of perceptions of current practice but different from that of preferences. Further studies are required to confirm and generalize our finding and to compare the views on identifiable and anonymised samples.

## Competing interests

The authors declare that they have no competing interests.

## Authors' contributions

Conceived the idea and designed study (MMH and MAQ), conducted literature review (EAG, MAQ), collected data (MAQ, HAH, EAG), performed statistical analysis (initial MAQ and MMH, revised MMH), wrote manuscript (MMH, EAG, MAQ). All authors read and approved the final manuscript.

## Pre-publication history

The pre-publication history for this paper can be accessed here:

http://www.biomedcentral.com/1472-6939/11/18/prepub

## Supplementary Material

Additional file 1 **Study questionnaires**. The six study questionnaires together with the introductory information given to participants. Click here for file
