# Supplementary material for: Saudi views on consenting for research on medical records and leftover tissue samples
Source: BMC Med Ethics. 2010 Oct 18;11:18. doi: 10.1186/1472-6939-11-18 (PMC2974743; doi:10.1186/1472-6939-11-18)
Supplement: Additional file 1 — Study questionnaires. The six study questionnaires together with the introductory information given to participants. [file 1472-6939-11-18-S1.DOC]

**Dear participant:**

Some medical research depends on studying the information contained in medical records or on leftover tissues (tissues that were previously obtained for clinical diagnostic or therapeutic purposes or other research purposes).

Such research do not usually provide direct benefits to those whose medical records or leftover tissues are studied. However, it could enrich medical knowledge and be the beginning of medical progress. On other hand, the information contained in medical records and leftover tissues are obtained for medical care purposes and are considered confidential. Patients may not wish that their private medical information or tissues be seen by others or used for other purposes without their permission.

The aim of the current study is to know your opinion. There are three sets of statements about consenting for the use of medical records in research and three sets of similar statements about consenting for the use of leftover tissues in research. The first set is on what you personally prefer, the second set is on what you think is the norm (the rules that should be followed), and the third set is on what you think best reflect the current practice at KFSH&RC. Each set presents you with seven options for consenting. You have to choose one.

**I- What I personally prefer:**

Medical Records: (choose one answer)

1) I prefer that the information in my medical records is not used except for my medical care.

2) I prefer that the information in my medical records is not used in medical research unless I gave my prior consent for each type of research.

3) I prefer that the information in my medical records is not used in medical research unless I gave a general consent for research.

4) It is ok to use the information in my medical records in medical research if approval of the research ethics committee was obtained; there is no need for my consent.

5) It is ok to use the information in my medical records in medical research without my consent or the approval of the research ethics committee as long as the researcher is affiliated with KFSH&RC.

6) It is ok to use the information in my medical records in medical research without my consent or the approval of the research ethics committee as long as the researcher is Saudi.

7) It is ok to use the information in my medical records in medical research without my consent or the approval of the research ethics committee as long as the researcher is scientifically competent and regardless of his/her nationality.

**II- What I personally prefer:**

Leftover Samples:( choose one answer)

1) I prefer that my leftover tissues are not used except for my medical care.

2) I prefer that my leftover tissues are not used in medical research unless I gave my prior consent for each type of research.

3) I prefer that my leftover tissues are not used in medical research unless I gave a general consent for research.

4) It is ok to use my leftover tissues in medical research if approval of the research ethics committee was obtained; there is no need for my consent.

5) It is ok to use my leftover tissues in medical research without my consent or the approval of the research ethics committee as long as the researcher is affiliated with KFSH&RC.

6) It is ok to use my leftover tissues in medical research without my consent or the approval of the research ethics committee as long as the researcher is Saudi.

7) It is ok to use my leftover tissues in medical research without my consent or the approval of the research ethics committee as long as the researcher is scientifically competent and regardless of his/her nationality.

**III- What I think is the norm (the rules that should be followed, regardless of what I personally prefer):**

Medical Records: (choose one answer)

1) I think that the information in medical records should not be used other than for the medical care of the corresponding patient.

2) I think that the information in medical records should not be used in medical research unless the patient gave prior consent for each type of research.

3) I think that the information in medical records should not be used in medical research unless the patient gave a general consent for research.

4) It is ok to use the information in medical records in medical research if approval of the research ethics committee was obtained; there is no need for patient consent.

5) It is ok to use the information in medical records in medical research without patient consent or the approval of the research ethics committee as long as the researcher is affiliated with KFSH&RC.

6) It is ok to use the information in medical records in medical research without patient consent or the approval of the research ethics committee as long as the researcher is Saudi.

7) It is ok to use the information in medical records in medical research without patient consent or the approval of the research ethics committee as long as the researcher is scientifically competent and regardless of his/her nationality.

**VI- What I think is the norm (the rules that should be followed, regardless of what I personally prefer):**

Leftover Tissues: (choose one answer)

1) I think that leftover tissues should not be used other than for the medical care of the corresponding patient.

2) I think that leftover tissues should not be used in medical research unless the patient gave prior consent for each type of research.

3) I think that leftover tissues should not be used in medical research unless the patient gave a general consent for research.

4) It is ok to use leftover tissues in medical research if approval of the research ethics committee was obtained; there is no need for patient consent.

5) It is ok to use leftover tissues in medical research without patient consent or the approval of the research ethics committee as long as the researcher is affiliated with KFSH&RC.

6) It is ok to use leftover tissues in medical research without patient consent or the approval of the research ethics committee as long as the researcher is Saudi.

7) It is ok to use leftover tissues in medical research without patient consent or the approval of the research ethics committee as long as the researcher is scientifically competent and regardless of his/her nationality.

**V- What I think reflects current practice at KFSH&RC (regardless of what I prefer personally or I think is the norm):**

Medical Records: (choose one answer)

1) The information in medical records is not used other than for the medical care of the corresponding patient.

2) The information in medical records is not used in medical research unless the patient gave prior consent for each type of research.

3) The information in medical records is not used in medical research unless the patient gave a general consent for research.

4) The information in medical records is used in medical research if approval of the research ethics committee was obtained; there is no need for patient consent.

5) The information in medical records is used in medical research without patient consent or the approval of the research ethics committee as long as the researcher is affiliated with KFSH&RC.

6) The information in medical records is used in medical research without patient consent or the approval of the research ethics committee as long as the researcher is Saudi.

7) The information in medical records is used in medical research without patient consent or the approval of the research ethics committee as long as the researcher is scientifically competent and regardless of his/her nationality.

**VI- What I think reflects current practice at KFSH&RC (regardless of what I prefer personally or I think is the norm):**

Leftover Tissue: (choose one answer)

1) Leftover tissues are not used other than for the medical care of the corresponding patient.

2) Leftover tissues are not used in medical research unless the patient gave prior consent for each type of research.

3) Leftover tissues are not used in medical research unless the patient gave a general consent for research.

4) Leftover tissues are used in medical research if approval of the research ethics committee was obtained; there is no need for patient consent.

5) Leftover tissues are used in medical research without patient consent or the approval of the research ethics committee as long as the researcher is affiliated with KFSH&RC.

6) Leftover tissues are used in medical research without patient consent or the approval of the research ethics committee as long as the researcher is Saudi.

7) Leftover tissues are used in medical research without patient consent or the approval of the research ethics committee as long as the researcher is scientifically competent and regardless of his/her nationality.
